# Status of comprehensive geriatric assessment in hospital settings: a cross-sectional survey

**DOI:** 10.3389/fmed.2025.1635751

**Published:** 2025-09-25

**Authors:** Hong Lyu, Yan Dong, Rui Chen, Yan Zeng, Jia Liu, Xinhong Song, Huihui Wang, Liqun Xing, Xin Zhang, Ying Wang

**Affiliations:** 1 Department of Geriatrics, Shandong Provincial Hospital Affiliated to Shandong First Medical University, Jinan, China; 2 Department of Gynecology, Shandong Provincial Hospital Affiliated to Shandong First Medical University, Jinan, China; 3 Property Supervision and Management Office, Shandong Provincial Hospital Affiliated to Shandong First Medical University, Jinan, China; 4 Department of Traumatic Orthopedics, Shandong Provincial Hospital Affiliated to Shandong First Medical University, Jinan, China; 5 Medical Insurance Office, Shandong Public Health Clinical Center, Jinan, China

**Keywords:** comprehensive geriatric assessment, older person, geriatric nurses, medical institution, aging

## Abstract

**Background:**

The Comprehensive Geriatric Assessment (CGA) serves as a crucial multidimensional instrument for optimizing care within aging populations. Despite its demonstrated benefits, there remain significant implementation gaps in China, particularly within Shandong Province. This study examines the current status, facilitators, and barriers to CGA implementation from the perspective of geriatric nurse specialists (GNS).

**Methods:**

A cross-sectional survey was administered to 200 GNS trained by the Shandong Nursing Association between 2018 and 2022. Data collection was conducted using a validated questionnaire grounded in the Unified Theory of Acceptance and Use of Technology (UTAUT) framework. Statistical analyses were performed using chi-square tests and odds ratio (OR), with significance set at *p* < 0.05.

**Results:**

CGA implementation was observed in only 50.50% of medical institutions. Key facilitators included Hospital level (secondary/tertiary: OR = 5.30, 95% CI: 2.29–12.25), Staff training (OR = 5.39, 95% CI: 1.75–16.56), Dedicated CGA personnel (OR = 3.41, 95% CI: 1.86–6.24), Interventions based on CGA results (OR = 7.34, 95% CI: 2.44–22.12). Unexpectedly, GNS certification (OR = 0.44, 95% CI: 0.21–0.91) appeared to impede implementation. The primary barriers identified were the time-intensive nature of the process (64%), insufficient involvement of multidisciplinary teams (62%), and the absence of insurance reimbursement (48%).

**Conclusion:**

The adoption of CGA in Shandong remains below optimal levels. To enhance implementation, it is imperative to develop policy-driven strategies that include integrating CGA into insurance reimbursement frameworks, standardizing digital workflows, expanding multidisciplinary teams, and addressing workforce shortages through targeted training initiatives.

## Background

The Comprehensive Geriatric Assessment (CGA) is a systematic approach designed to thoroughly evaluate the health status of older person. Its objective is to identify physical, psychological, and social health issues, as well as potential health risks, through a multidimensional and interdisciplinary team assessment. This process serves as a foundation for formulating individualized care or treatment plans aimed at enhancing the quality of life for older person and preventing or mitigating the occurrence of complications (1). It is one of the core technologies in modern medicine and is an effective means of screening for geriatric syndromes. CGA has important guiding significance and clinical application value for the diagnosis and treatment of acute geriatric diseases, as well as intermediate care, long-term care, hospice care, and chronic disease prevention and control in the late acute and sub-acute stages. CGA can significantly promote the functional status of older person, improve their quality of life and health, and reduce the adverse health effects of population aging (2–4).

Aging in China has further aggravated in recent years. By the end of 2020, the older population aged 60 years and above in China had reached 267 million, accounting for 18.9% of the total population (5). It is predicted that the number of people with disabilities in China will exceed 70 million by 2030.

Shandong is the second most populous Province in China. A 3-month geriatric nurse specialist training program was launched by the Shandong Nursing Association in 2018 to improve the professional service abilities of geriatric nursing practitioners and meet the demands of older person. The geriatric nurse specialists should play an important leading role in the multidisciplinary team of CGA (6, 7).

However, CGA was not thoroughly implemented in China, with only 63.6% conducting CGA in East China, particularly in Shandong Province, which is the northernmost part of East China (8). At present, there is no research on the status and difficulties of CGA in Shandong Province, especially from the perspective of geriatric nurses.

This study aimed to investigate the current situation and difficulties in CGA implementation in medical institutions by surveying geriatric nurse specialists trained by the Shandong Nursing Association using a cross-sectional design, to provide optimization suggestions for the implementation of CGA and a basis for decision-making by superior departments.

## Methods

### Participants

A list of 636 geriatric nurse specialists over four training sessions from 2018 to 2022 was obtained through the Shandong Nursing Association, and participants were selected using simple random sampling. Specifically, training program was suspended for the entire year of 2020 due to the COVID-19 pandemic. According to the sample size calculation formula $N=\frac{\mu 2\times p\times (1-p)}{d2}$ (confidence level, 95%; *μ*, 1.96; *p* = 0.871), the sample size was determined to be 172. Considering a 95% response rate, the minimum sample size was set to be 181.

Inclusion criteria:

(1) Registered nurse with a college degree or higher.
(2) Minimum 2 years of geriatric care experience in medical institutions.
(3) Certified geriatric nurse specialist.
(4) Voluntarily participation with signed informed consent.

Exclusion criteria:

(1) Failure to complete >80% of the questionnaire items.
(2) Withdrawal during data collection period.

The study was approved by the Ethics Committee of Shandong Provincial Hospital Affiliated to Shandong First Medical University (SWYX: NO.2021**-**096).

### Research tools

A questionnaire grounded in the Unified Theory of Acceptance and Use of Technology (UTAUT) framework, as developed by Huang et al. (9), is employed to assess the ease of use, perceived usefulness, and the social and facilitating conditions influencing users, with the aim of predicting the adoption of these technological solutions. The survey questionnaire consisted the following parts (8, 10, 11):

1. Demographic information. Gender, age, professional title, educational background, type of medical institution, nature of medical institution, and level of medical institution.
2. Information on learning knowledge of CGA. Level of CGA Knowledge Awareness, Participation in CGA Training, Format of CGA Training Received, Prior Experience in CGA Practice.
3. Information on CGA carried out by the medical institution.

Hospital Level, Received CGA Training, Prior CGA Experience, Staff specifically assigned for CGA, CGA Report Generation Capability, Interventions based on assessment results, Geriatric nurse specialist certification.

4. Problems and difficulties in implementing CGA.

(1) The evaluation is time-consuming, with a large number of questionnaire tools and a cumbersome process.
(2) Multidisciplinary teams are not involved enough in CGA.
(3) CGA does not charge, and medical evaluation is not highly motivated.
(4) Old people coexist with many diseases, complex and changeable.
(5) CGA is generally not well understood by medical staff.
(8) Older people are not aware of the importance of CGA.

### Data collection and quality control

Four professionally trained geriatric specialist nurses distributed and collected electronic questionnaires, respectively, among four certified geriatric specialist nurse groups who had completed training and obtained qualifications. 20% of entries were cross-checked by a second investigator against original records. A total of 200 electronic questionnaires were distributed and recovered, all of which were effective. The recovery rate was 100%, and the effective rate was 100%.

### Statistical methods

Statistical software (Statistical Package for the Social Sciences version 25.0) was used for data processing, with normally distributed continuous data expressed as the mean ± standard deviation. The enumeration data are expressed as frequency and percentage, and the chi-squared was performed for intergroup comparisons. (1) For the chi-squared test of the fourfold table, if the theoretical number was *T* < 5 but *T* ≥ 1 and *n* ≥ 40, the continuity correction chi-squared was used for testing. (2) We also implemented a chi-squared test for R × C tables by column merging.

All statistical tests were two-sided, and the significance level was set at *p* < 0.05. Participants were divided into the carrying out group and the non-carrying out group based on the situation of conducting CGA to analyze the statistical differences in the incidence of carrying out CGA among the groups with different characteristics. We used odds ratio (OR) and 95% confidence interval (CI) to indicate the strength of the association between carrying out CGA and exposure in Geriatric nurse specialists.

## Results

### Basic information

This study included 200 geriatric nurse specialists with a mean age of 34.38 ± 5.99 years. Most participants (82.00%) were females. Among them, 127 (69.00%) were employed in general hospitals, 161 (80.50%) in secondary or tertiary hospitals. Additionally, 160 (80.00%) had a bachelor’s degree or higher, and 135 (67.50%) held an intermediate professional title or higher (Table 1).

**Table 1:** Demographic characteristics of geriatric nurse specialists (*n* = 200).

Item		Frequency	Percentage (%)
Gender	Male	36	18.00
	Female	164	82.00
Age (years)	<29	32	16.00
	30–39	98	49.00
	40–49	52	26.00
	≥50	18	9.00
Years of experience	<9	60	30.00
	10–19	86	43.00
	20–29	35	17.50
	≥30	19	9.50
Professional title	Junior	65	32.50
	Intermediate	94	47.00
	Senior	41	20.50
Education level	Associate degree or lower	40	20.00
	Bachelor’s degree	146	73.00
	Master’s degree or higher	14	7.00
Hospital type	General hospital	138	69.00
	Geriatric specialty hospital	29	14.50
	Traditional Chinese medicine specialty hospital	17	8.50
	Other healthcare institutions	16	8.00
Hospital level	Tertiary hospitals	115	57.50
	Secondary hospitals	46	23.00
	Primary hospitals	39	19.50

### Information on learning knowledge of CGA

Additionally, 89.00% of the geriatric nurse specialists included in the study had undergone training in CGA, 72.00% possessed work experience in the field. The primary means through which they acquired pertinent knowledge were academic conferences (80.00%), scenario simulation (55.50%), and online courses (52.50%) (Table 2). The average level of knowledge about CGA was determined to be 3.90 ± 0.97 by the Likert five-level scoring method.

**Table 2:** Information on learning knowledge of CGA (*n* = 200).

Item		Frequency	Percentage (%)
Level of CGA knowledge awareness	Uninformed	5	2.50
	Minimally informed	12	6.00
	Moderately informed	38	19.00
	Fairly well-informed	88	44.00
	Very well-informed	57	28.50
Participation in CGA training	No	22	11.00
	Yes	178	89.00
Format of CGA training received	Academic conferences	160	80.00
	Online courses	111	55.50
	Scenario simulation	105	52.50
	Other formats	5	2.50
Prior experience in CGA practice	No	56	28.00
	Yes	144	72.00

### Status of carrying out CGA in hospitals

A total of 50.50% of medical institutions conducted a CGA in the geriatric ward on arrival to the hospital, with 62.50% of these institutions employing dedicated personnel for this purpose. Most of CGA was conducted by nurses (40.50%) and multidisciplinary teams (35.50%). Most staff (80.5%) involved in the CGA possessed specialized qualifications. CGA was primarily (85.50%) conducted within inpatient wards, with only 13.50% of outpatients offering this service. Furthermore, a significant proportion (48.00%) of CGA medical service were not covered by medical insurance. Most medical institutions conducted a CGA utilizing electronic platforms (80.50%), including general medical assessment, assessment of mental and psychological status, physical function assessment, environmental health assessment, assessment of social behavior ability, etc. (Table 3).

**Table 3:** Information on CGA carried out by the medical institution (*n* = 200).

Item		Frequency	Percentage (%)
Staff specifically assigned for CGA	Yes	125	62.50
Medical workers involved in CGA	Nurse	81	40.50
	Multidisciplinary team	71	35.50
	Doctor	48	24.00
Geriatric nurse certification required	In-hospital level	91	45.50
	Provincial level and above	70	35.00
	No	39	19.50
Job categories of CGA	Clinical nursing	116	58.00
	Medical technology	54	27.00
	Administrative management	21	10.50
	Others	9	4.50
In-hospital settings for CGA	Outpatient and wards	94	47.00
	Wards	77	38.50
	Outpatient	27	13.50
	Other	2	1.00
Carrier of CGA	Paper and electronic	87	43.50
	Electronic	74	37.00
	Paper	39	19.50
Content of performing CGA	General medical assessment	147	73.50
	Assessment of mental and psychological status	143	71.50
	Physical function assessment	141	70.50
	Environmental health assessment	117	58.50
	Assessment of social behavior ability	117	58.50
Age of patients for CGA	60 years or older	100	50.00
	65 years or older	79	39.50
	70 years or older	16	8.00
	Other	5	2.50
Medical insurance reimbursement	Can not charge	96	48.00
	Partial or all charge	104	52.00

### Facilitators of implementing CGA

Factors such as secondary hospital or above (OR = 5.300, 95%CI = 2.293–12.249), prior CGA experience (OR = 3.602, 95%CI = 1.846–7.026), received CGA training (OR = 5.389, 95%CI = 1.753–16.563), dedicated CGA staff (OR = 3.409, 95%CI = 1.863–6.239), CGA report generation capability (OR = 1.843, 95%CI = 1.020–3.329), and assessment-based interventions (OR = 7.339, 95%CI = 2.435–22.123) significantly contribute to comprehensive geriatric assessment implementation. Surprisingly, geriatric specialist nurse qualifications (OR = 0.436, 95%CI = 0.209–0.909) do not yet appear to be a prerequisite for implementing comprehensive geriatric assessments (Table 4).

**Table 4:** Facilitators of implementing comprehensive geriatric assessment (CGA).

Variables		Not conduct CGA	Conducting CGA	*X* ^2^	*P*	OR	95%CI
Hospital level				17.428	<0.001	5.300	2.293–12.249
Primary hospital		31	8				
Secondary hospital or above		68	93				
Received CGA training				10.328	0.001	5.389	1.753–16.563
No		18	4				
Yes		81	97				
Prior CGA experience				14.962	<0.001	3.602	1.846–7.026
No		40	16				
Yes		59	85				
Staff specifically assigned for CGA				16.430	<0.001	3.409	1.863–6.239
No		51	24				
Yes		48	77				
CGA report generation capability				4.147	0.042	1.843	1.020–3.329
No		41	28				
Yes		58	73				
Interventions based on assessment results				15.901	<0.001	7.339	2.435–22.123
No		23	4				
Yes		76	97				
Geriatric nurse specialist certification				5.065	0.024	0.436	0.209–0.909
Primary hospital	No	4	0	0.176	0.675	0.296	1.082–1.552
	Yes	27	8				
Secondary hospital or above	No	9	26	5.004	0.025	0.393	0.171–0.906
	Yes	59	67				

### Barriers in the implementation of CGA

Time-consuming, insufficient participation of multidisciplinary teams, free assessment, old people coexisting with many diseases, unawareness of the importance of CGA, and employee knowledge deficits were the main reasons that hindered the implementation of CGA (Table 5).

**Table 5:** The barriers in CGA implementation (*n* = 200).

Item	Frequency	Percentage (%)
The evaluation is time-consuming, with a large number of questionnaire tools and a cumbersome process, which is not easy to be accepted by beginners	128	64.0
Multidisciplinary teams are not involved enough in CGA	124	62.0
CGA does not charge, and medical evaluation is not highly motivated	110	55.0
Old people coexist with many diseases, complex and changeable	110	55.0
CGA is generally not well understood by medical staff	103	51.5
Older people are not aware of the importance of CGA	96	48.0

## Discussion

This cross-sectional study provides new evidence for the promotion of CGA in our province by analyzing the current status and implementation facilitators and barriers from the perspective of geriatric nurses.

### The implementation extent of CGA

The rate of implementation of CGA in medical institutions within Shandong Province was found to be 50.50%, which was consistent with the result of 55.1% in a cross-sectional study conducted in 390 medical institutions across 31 provinces in China (8). The rate of CGA implementation might have been influenced by national policies focusing on geriatric care demand assessment (12) and the establishment of geriatric-friendly hospitals (13). However, there remains a significant scope for the promotion of CGA in geriatric medicine and outpatient clinics.

CGA has been implemented in multiple countries including the United States, Germany, Japan, Australia, and South Korea. It is now widely applied in outpatient geriatric clinics, hospitalized elderly patients, and home-based care settings, demonstrating positive impacts on health status and long-term prognosis. Nonetheless, challenges remained including insufficient standardization, workforce shortages, high economic costs, low participation willingness among older adults, inadequate professional training, and regional disparities (14–16).

Geriatric nurse specialists in our province have received structured theoretical and practical instruction pertaining to the CGA. It is imperative for them to amass further practical experience within clinical settings, emphasize the significance of communication and coordination within multidisciplinary teams, and fully exploit the potential of CGA technology to enhance patient outcomes. There are statistical differences between different medical institutions in the Job Categories of CGA and whether to form an assessment report, hence, hospital management departments are obligated to enhance the management of CGA and facilitate the presentation of CGA results through joint efforts of network departments and other relevant departments.

### Facilitators of implementing CGA

#### Institutional capacity

The facilitating effect of secondary/higher-level institutions on CGA implementation may stem from China’s 2020 national policy (“Work Plan for Establishing Age-Friendly Healthcare Institutions”), which explicitly requires secondary and above hospitals to deliver CGA. This underscores the critical role of resource infrastructure in implementing complex geriatric care models. Secondary and tertiary hospitals should be developed into designated CGA training centers to provide technical guidance for CGA in community healthcare settings.

#### Human resource factors

CGA-trained personnel, prior assessment experience, and dedicated staffing significantly facilitate CGA implementation. This aligns with King et al.’s findings (17) demonstrating that proficient clinicians enhance CGA execution through comprehensive explanations of patients’ medical, psychological, and functional status. Staff training (OR = 5.389) exerts the strongest human-resource impact, highlighting that specialized competency is non-negotiable for effective CGA delivery. Clinicians with CGA experience (OR = 3.602) are more likely to support adoption, emphasizing the value of practical exposure in building confidence. Dedicating staff (OR = 3.409) exclusively to CGA increases CGA adoption demonstrating that focused resource allocation is essential to sustainable implementation. Human factors collectively form the most potent cluster. Without trained, experienced, and dedicated staff, even well-resourced institutions struggle. Subsequent research ought to prioritize human capital development via institution of dedicated CGA positions with structured career progression frameworks.

#### Technical capabilities

The capability to generate CGA reports facilitates CGA implementation. Research by Sujker et al. (18) demonstrates that digitizing the CGA process, coupled with reliable and user-friendly assessment tools, streamlines data collection and subsequent analysis, thereby positively influencing CGA adoption. Post-assessment generation of a unified report effectively highlights clinically relevant findings across the physical, psychological, and social domains of older adults, guiding physicians in developing targeted interventions. Despite statistical significance, this factor shows the lowest OR among all predictors. Its contribution is likely supplementary that automated reports optimize workflows but remain contingent upon human expertise for data interpretation and clinical decision-making. This is consistent with prior research showing that digitization enables, yet does not singularly determine CGA adoption (19). We recommend that healthcare institutions adopt digital CGA platforms during training but prevent premature technology dependence prior to resolving workforce shortages.

#### Outcome-oriented practices

Assessment-Based Interventions (OR = 7.339) emerges as the strongest predictor of CGA adoption. When CGA findings directly inform tailored interventions, clinicians recognize actionable clinical value. Subsequent efforts must prioritize developing CGA pathways centered on interventions, ensuring assessment findings prompt predetermined responses, with systematic monitoring and feedback on outcomes to sustain the older patients engagement.

### Barriers of CGA in hospital settings

#### Operational challenges

In total, 64.00% of geriatric nurse specialists considered a plethora of items and scales available for conducting a thorough assessment of the older person, at the cost of significant time and human resources. Meanwhile, only 65.50% of these institutions were able to generate CGA reports for older person. Consequently, it is imperative that assessment tools tailored for the older person population offer a more comprehensive evaluation, while simultaneously addressing the challenges of simplicity and expediency (20, 21). The optimization of the CGA workflow and management system is also important. (22) The integration of artificial intelligence-driven triage systems to automate initial evaluations, alongside the adoption of the World Health Organization’s Integrated Care for Older People (ICOPE) toolkit to standardize assessment protocols, represents an effective strategy for minimizing time expenditure in CGA (23, 24).

#### MDT integration

In this study, geriatric nurse specialists posited that the insufficient engagement of multidisciplinary teams (62.00%) impedes the effective implementation of comprehensive geriatric assessments. These teams typically comprise geriatricians, geriatric nurses, clinical nutritionists, occupational therapists, psychologists, pharmacists, and social workers, either in a partial or full capacity. The involvement of multi-agency teams in geriatric assessment and care delivery is influenced by variations in organizational culture, psychological models of service, divergent expectations of job responsibilities, and the potential for work duplication, all of which impact the establishment and longevity of partnerships (17, 20). In addition, the absence of direct communication between geriatric assessment staff and general practitioners made the applicability of these assessments uncertain.

In addition, according to Chadborn et al. (25), a critical review of nursing home CGA revealed that its successful implementation requires three key elements: the formulation of a care plan, a structured comprehensive assessment, and the pursuit of patient-centered goals, all of which require the collaboration of a multidisciplinary team (MDT). Hence, it is helpful to establish dedicated roles to synchronize MDT workflows of CGA and develop digital collaboration platforms to facilitate MDT consultation (26).

#### Financial constraints

Currently, the charging predicament of CGA in Shandong Province is not promising. Approximately 48.00% of institutions cannot levy fees for a portion of the evaluation project, which affected the enthusiasm of patients to receive CGA. It is imperative to enhance collaboration with medical insurance and other relevant departments, bolstered by government and social support, to augment assistance and investment in CGA (27). In recent years, with the issuance of a sequence of policy documents (12, 28) under the National Healthy China Action, we advocate for the integration of CGA-specific billing codes into provincial DRG payment frameworks.

#### Staff competency

The unskilled staff (51.50%) is also a factor that hinders the implementation of CGA. Competent personnel play a crucial role in the successful execution of CGA. The staff’s comprehensive elucidation of the patients’ medical, psychosocial, and functional states significantly contributed to enhancing their health literacy and the probability of older person embracing the suggested services (17). In intricate cases, the staff’s capacity to anticipate the patient’s requirements and effectively coordinate care among various providers is highly esteemed by patients (29). In addition, research has shown that the cognitive level of medical staff can promote the implementation of CGA (30). While geriatric nurse certification does not facilitate the implementation of CGA, especially in secondary and above general hospitals, the involvement of specialized personnel remains essential for its execution. This ostensibly paradoxical observation may be explained by the acute shortage of trained geriatric nurses in Shandong province or by a lack of awareness among administrators about the significance of CGA. As a result, healthcare institutions across various levels have not yet allocated dedicated geriatric nurses to undertake this responsibility. We recommend administrators and medical staffs participate programs as competency-based training of CGA specialists through geriatric nursing certification or elderly ability assessment practitioner to elevate CGA knowledge.

#### Patient factors

Patients lacking awareness of the importance of CGA (48.00%) is also a barrier. Healthcare providers encounter challenges when attempting to persuade patients of the preventive advantages of CGA. Patients frequently possess varying interpretations of health issues and may not perceive preventive services as personally valuable (31, 32). Apprehensions about data security (20) impede older person’ ability to establish trust and actively participate in CGA services. Consequently, it is imperative for healthcare professionals to actively educate patients regarding the preventive advantages associated with the CGA and to develop and implement health programs aimed at enhancing patient awareness of CGA (33).

## Limitations

First, the study samples were selected from a single province. Therefore, the generalization of the study findings would be inappropriate. Second, the present study was limited by cross-sectional analyses, and no information was available on the health outcomes of patients receiving CGA; therefore, the impact of comprehensive geriatric assessment on patient health outcomes could not be determined. Finally, having GNS perspectives may not fully represent institutional barriers or other multiple disciplinary team perspectives, future studies could employ mixed-methods designs and health system frameworks to explore implementation barriers.

This study reveals moderate CGA implementation (50.5%) in Shandong hospitals. Key facilitators include higher institutional capacity, specialized staff training, dedicated personnel, and intervention-focused practices. Notably, geriatric nurse certification did not enhance implementation, suggesting workforce allocation or prioritization issues. Major barriers were time-intensive processes, poor multidisciplinary coordination, and lack of insurance coverage.

To advance CGA adoption, we recommend: (1) Integrating CGA billing into provincial DRG frameworks; (2) Implementing digital tools to streamline workflows; (3) Enhancing team coordination through dedicated roles and digital platforms; and (4) Strengthening staff training programs. Future efforts should prioritize patient education and multi-center studies to address regional disparities.

## Data Availability

The original contributions presented in the study are included in the article/supplementary material, further inquiries can be directed to the corresponding author.
