# Correction: Status of comprehensive geriatric assessment in hospital settings: a cross-sectional survey

**DOI:** 10.3389/fmed.2025.1742940

**Published:** 2025-11-26

**Authors:** Hong Lyu, Yan Dong, Rui Chen, Yan Zeng, Jia Liu, Xinhong Song, Huihui Wang, Liqun Xing, Xin Zhang, Ying Wang

**Affiliations:** 1 Department of Geriatrics, Shandong Provincial Hospital Affiliated to Shandong First Medical University, Jinan, China; 2 Department of Gynecology, Shandong Provincial Hospital Affiliated to Shandong First Medical University, Jinan, China; 3 Property Supervision and Management Office, Shandong Provincial Hospital Affiliated to Shandong First Medical University, Jinan, China; 4 Department of Traumatic Orthopedics, Shandong Provincial Hospital Affiliated to Shandong First Medical University, Jinan, China; 5 Medical Insurance Office, Shandong Public Health Clinical Center, Jinan, China

**Keywords:** comprehensive geriatric assessment, older person, geriatric nurses, medical institution, aging

An incorrect number was provided for the funder Shandong Province Science and Technology Projects of Medicine and Health. The correct number is 2023BJ000035.

The original version of this article has been updated.

